# “One Pot” Enzymatic Synthesis of Caffeic Acid Phenethyl Ester in Deep Eutectic Solvent

**DOI:** 10.3390/biom15020181

**Published:** 2025-01-27

**Authors:** Maria Roberta Tripon, Camelia Tulcan, Simona Marc, Dorin-Dumitru Camen, Cristina Paul

**Affiliations:** 1 Faculty of Engineering and Applied Technologies, University of Life Sciences “King Mihai I” from Timișoara, Calea Aradului No. 119, 300645 Timișoara, Romania; roberta.tripon@usvt.ro (M.R.T.); dorincamen@usvt.ro (D.-D.C.); 2 Faculty of Veterinary Medicine, University of Life Sciences “King Mihai I” from Timișoara, Calea Aradului No. 119, 300645 Timișoara, Romania; simona.marc@usvt.ro; 3 Department of Applied Chemistry and Engineering of Organic and Natural Compounds, Faculty of Chemical Engineering, Biotechnologies and Environmental Protection, Politehnica University Timisoara, Vasile Pârvan No. 6, 300223 Timișoara, Romania

**Keywords:** caffeic acid phenethyl ester (CAPE), enzymatic synthesis, deep eutectic solvent, reaction optimization

## Abstract

Caffeic acid phenethyl ester (CAPE) represents a valuable ester of caffeic acid, which, over time, has demonstrated remarkable pharmacological properties. In general, the ester is obtained in organic solvents, especially by the esterification reaction of caffeic acid (CA) and 2-phenylethanol (PE). In this context, the purpose of this study was the use of the “one pot” system to synthesize CAPE through biocatalysis with various lipases in a choline-chloride-based DES system, employing the “2-in-1” concept, where one of the substrates functions as both reactant and solvent. The synthesis process of CAPE is contingent on the molar ratio between CA and PE; thus, this factor was the primary subject of investigation, with different molar ratios of CA and PE being studied. Furthermore, the impact of temperature, time, the nature of the biocatalyst, and the water loading of the DES system was also examined. This ‘green’ synthesis method, which has demonstrated encouraging reaction yields (%), could secure and maintain the therapeutic potential of CAPE, mainly due to the non-toxic character of the reaction medium.

## 1. Introduction

Caffeic acid phenethyl ester is a natural derivate of caffeic acid, primarily isolated from propolis, with the molecular formula of C_17_H_16_O_4_. Its ACD/IUPAC name is 2-phenylethyl (2E)-3-(3,4-dihydroxyphenyl)acrylate, but it is also known as phenethyl caffeate or CAPE [1]. First identified by Grunberger et al. in 1988 [1], CAPE is a polyphenol with hydroxyl groups in the catechol ring which are responsible for its notable role in numerous biological processes. In the early 1990s, researchers began to investigate and confirm the anti-inflammatory and antineoplastic properties of propolis. They discovered that this ester was one of the primary biologically active components of propolis, and is responsible for many of its well-known benefits [2]. Since then, various studies have explored the therapeutic potential of CAPE, demonstrating that it possesses antioxidant [3,4,5], anti-inflammatory [6,7,8], anticancer [9,10,11], antiviral [7,12,13], antibacterial, and antifungal [14,15,16] properties, among many others.

Generally, caffeic acid phenethyl ester (CAPE) is obtained through enzymatic biocatalysis. This synthesis process is carried out by adding phenethyl alcohol to a solution of caffeic acid in an organic solvent at a controlled temperature and pressure. In this process, the enzyme acts as a catalyst, shortening the reaction time and creating a high-quality product. To date, most pioneering publications have reported that the yields of caffeic acid phenethyl ester (CAPE) obtained from caffeic acid (CA) and 2-phenylethanol (PE) through esterification catalyzed by Novozym 435 in isooctane at approximately 70 °C reached considerable levels. However, optimization of these reactions is still desired, especially regarding the degree of stability [17]. Although the current literature refers to the obtaining of CAPE through esterification and transesterification processes involving enzymes such as *Candida antarctica* lipase B, optimization of this process is necessary to obtain satisfactory conversion percentages. Also, great attention is paid to the degree of product stability, as well as the reuse of enzymatic models. Moreover, in order to maintain the pharmaceutical properties of the ester, the possibility of using chemical synthesis methods involving alternative reaction media to classic organic solvents is mentioned. These alternatives refer to green solvents that do not have a negative impact at the tissue or cellular level. This group of solvents includes both ionic liquids and eutectic solvents (deep eutectic solvents or DES). As for ionic liquids, it is well known that many of them are (eco)toxic and harmful to the environment [18,19]. Thus, the most promising alternative is represented by the use of deep eutectic solvents (DES). In addition to being easy to prepare and having low costs and reduced volatility and flammability, it has been demonstrated that these solvents possess superior biodegradability compared to ionic liquids, with many of them being derived from natural molecules [18]. Some of the most significant properties of DES, including their ability to enhance ecological sustainability and generate economic advantages, are summarized in Table 1.

In this context, the aim of the present study is represented by caffeic acid phenethyl ester (CAPE) synthesis in deep eutectic solvents, a promising class of solvents with intriguing physical properties, which meet the criteria for green solvents. The synthesis of CAPE in DES was based on the “2 in 1” concept, in which one of the substrates (caffeic acid) acts both as a reactant and as a solvent, and CAPE is obtained in a “one pot” system of choline-chloride-based DES. The reaction yields achieved in this study are encouraging, and our results show that it is possible to produce this valuable ester in a “green” and non-toxic reaction environment.

## 2. Materials and Methods

Lipase B from *Candida antarctica*, recombinant, expressed in *Aspergillus niger*, was immobilized on acrylic resin (Novozyme 435). The commercial preparation GF CalB-IM^TM^ (generously donated by Genofocus, Daejeon, Republic of Korea) contains lipase from *Candida antarctica* B, produced by the fermentation of genetically modified microorganisms, adsorbed onto a microporous ion exchange resin. The lipase from *Pseudomonas stutzeri* was from Meito Sangyo, Nagoya, Japan. Caffeic acid was acquired from Sigma-Aldrich, Saint Louis, USA, and the CAPE standard (purity ≥ 98%) from TCI Chemicals, Tokyo, Japan. Methyl caffeate (purity ≥ 97.5%) was obtained from Thermo Fisher Scientific, Kandel, Germany. 2-phenylethanol (purity ≥ 99%) was obtained from Carl Roth GmbH, Karlsruhe, Germany. The solvents isooctane (~99%), acetone (~99%), acetonitrile (~99%), tetrahydrofuran (~99%), methanol (purity ≥ 99%), and hexane (~99%) were obtained from Merck, Darmstadt, Germany, and methyl-tetrahydrofuran was purchased from Sigma-Aldrich, Saint Louis, MO, USA.

The silane precursors used for the sol–gel entrapment of the native lipase from *Pseudomonas stutzeri* were octyl-trimethoxysilane (OcTMOS) from Fluka, tetra-methoxysilane (TMOS) from Acros Organics, New Jersey, USA, and 3-glycidoxypropyl-trimethoxysilane (GPTMS) 99+% (product code SIG5840.1), purchased from Gelest, Morrisville, PA, USA.

### 2.1. The Methodology of Immobilization by Entrapment in a Sol–Gel of Lipase from Pseudomonas stutzeri

The immobilization of lipase from *P. stutzeri* was carried out using two different methods based on our previous studies: Method 1—when octyltrimethoxysilane (OcTMOS) and tetramethoxysilane (TMOS) were used as silane precursors in a 1:1 molar ratio, PsL-SG1 enzymatic preparate was obtained [27]. Method 2—when glycidoxypropyltrimethoxysilane-3 (3-GoPrTMOS) and tetramethoxysilane (TMOS) were used as silane precursors in a 1:1 molar ratio, the PsL-SG2 enzymatic preparate was obtained [28]. The enzymes thus immobilized, along with the native ones (obtained from various producers), were used to facilitate the esterification process through the enzymatic biocatalysis of caffeic acid phenethyl (CAPE).

### 2.2. The Methodology of Enzymatic Synthesis of CAPE in Organic Solvents

In screw-capped glass tubes, 10.0 ± 0.5 mg of caffeic acid was accurately weighed, to which 2-phenylethanol was added at a molar ratio of acid:alcohol of 1:92. A 50.0 ± 0.5 mg amount of enzyme (commercial lipase isolated from colonies of *Aspergillus niger* and immobilized, AnL-IM) and the corresponding solvent were added to the tubes (Table 2).

The used solvents were acetone, acetonitrile, tetrahydrofuran, methyl-tetrahydrofuran, hexane, and isooctane. In the context of organic synthesis, the polarity of a solvent is conventionally quantified by log P, wherein P denotes the partition coefficient of the solvent between octanol and water. The log P values of the used solvent are presented in Table 2 [29,30,31].

**Figure 1:**
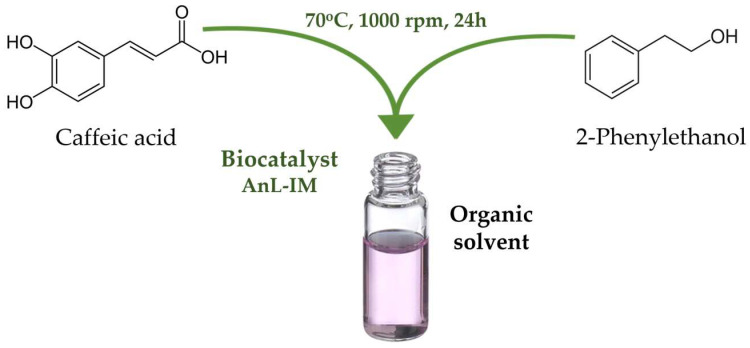
Graphical workflow of CAPE synthesis by lipase biocatalysis in organic solvents.

The sample thus prepared was incubated at 70 °C under continuous shaking at 1000 rpm for 24 h (Figure 1). The primary chromatographic determination (HPLC) was conducted at 24 h, while the stability of the sample was further monitored through additional measurements performed at 48 and 72 h. All reactions were conducted in duplicate, with sampling also undertaken in duplicate.

### 2.3. The Methodology of Enzymatic Synthesis of CAPE in Deep Eutectic Solvent

In order to maintain the pharmacological properties of CAPE and to reduce the toxicity associated with the use of organic solvents, the enzymatic synthesis of the ester was carried out in a eutectic medium formed in “one-pot” from the reaction of choline chloride, [(CH_3_)_3_NCH_2_CH_2_OH]^+^Cl^−^, and caffeic acid, C_9_H_8_O_4_. In this study, we proposed 13 esterification reactions, as shown in Table 3. All reactions were conducted in duplicate, with sampling also undertaken in duplicate.

**Figure 2:**
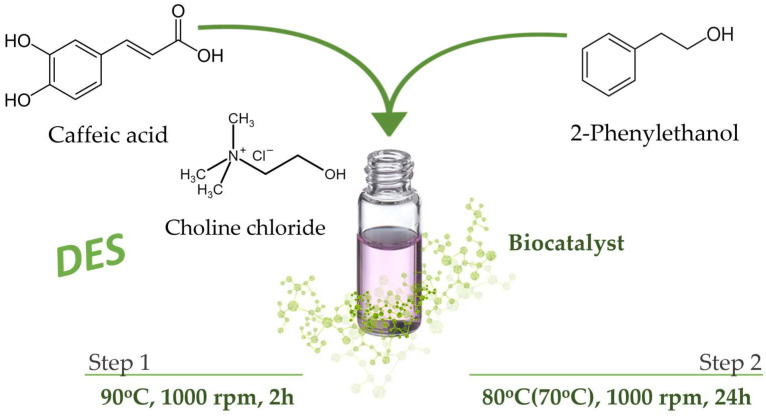
Graphical workflow of CAPE synthesis by lipase biocatalysis in deep eutectic solvent.

A total of 50.0 ± 0.5 mg caffeic acid and 77.5 ± 0.5 mg choline chloride were accurately weighed into 2 mL Eppendorf vials, ensuring a molar ratio of CA:ChCl of 1:2. The samples thus prepared were heated in an oil bath at 90 °C under continuous stirring at 1000 rpm for 2 h, producing a brownish complex of semi-viscous consistency. Following this step, the calculated volume of 2-phenylethanol (PE) was added to the determined amount of enzyme, and the mixture thus formed was placed in a thermostat at 80 °C (in the case of reactions from DesR_1_ to DesR_5_) and 70 °C (in the case of reactions from DesR_6_ to DesR_11_) under continuous stirring at 1000 rpm for 24 h (Figure 2). For DesR_1_, modifications of the general method were applied. The caffeic acid mass was reduced to 10.0 ± 0.5 mg, and the choline chloride mass was equal to 15.5 mg. The primary chromatographic determination (HPLC) was conducted at 24 h, while the stability of the sample was further monitored through additional measurements performed at 48 and 72 h.

### 2.4. The Methodology of Enzymatic Synthesis of CAPE in Deep Eutectic Solvent with Water Addition

Considering the omnipresence of water and the hygroscopic nature of eutectic solvents, many specialized studies suggest adding water to eutectic solvents in order to tune their properties [32,33,34,35]. The protocol implemented was based on the eutectic solvent method and was conducted with different degrees of water loading in the reaction mixture (1, 2.5, and 5%). The process consisted of 3 reactions, based on the method principle used for DesR_5_. The molar ratio of caffeic acid (CA) to choline chloride (ChCl) was 1:2 as the caffeic acid (CA) and 2-phenylethanol (PE) molar ratio was 1:5. For each reaction, 50 ± 0.5 mg of immobilized *A. niger* lipase (AnL-IM) was used. The water addition percentage was as follows: 1% for DesRW_1_, 2.5% for DesRW_2_, and 5% for DesRW_3_ (Table 4). All reactions were conducted in duplicate, with sampling also undertaken in duplicate.

This water addition was performed in order to facilitate the homogenization process between caffeic acid and choline chloride, with the water acting as a medium that promotes the dissolution of caffeic acid and also making it more compatible with choline chloride, which is hygroscopic and readily dissolves in water.

### 2.5. Chromatographic Analysis

The amount of ester obtained in the esterification reactions was assayed by an HPLC system (Jasco Inc., Tokyo, Japan) equipped with a UV-VIS detector (UV-2070 Plus) set at a 330 nm wavelength. The separation was performed with the use of an RP-18 (LiChrospher^®^ from Merck, Darmstadt, Germany) column with a pore diameter of 5 μm and 24 cm × 4.6 mm size. The column thermostat was set at a constant temperature of 25 °C throughout the analysis. The volume of the injected samples was 2 μL. The used mobile phase was a mixture of methanol and water, in a MeOH:H_2_O ratio of 80:20, applied at a flow rate of 1 mL/min. The mixture was supplemented with 0.1% formic acid to ensure the quality of the chromatographic peak shape.

The initial step involved the preparation of a calibration curve for the quantitative analysis of caffeic acid phenethyl ester. The CAPE standard (purity ≥ 98%) was used, and methanol was chosen as the solvent, with a total volume of 1 mL. The internal standard (IS) was methyl caffeate, in a solution with a concentration of 1 mg/mL. A stock solution of CAPE was prepared with a concentration of 1 mg/mL. Into seven chromatographic vials, precise volumes of caffeic acid phenethyl ester standard were weighed and the internal standard was added. Following the plotting of the calibration curve, a high correlation coefficient of R^2^ = 0.9997 and the following linear equation were obtained: y = 0.8228x + 0.0393 between area CAPE/area IS and mass CAPE/mass IS.

The ester yield and catalytic efficiency of the biocatalysts were determined based on the HPLC data. The ester yield (%) was calculated as the ratio between the amount of ester obtained after 24 h of reaction and the theoretical amount of ester. The catalytic efficiency of the biocatalysts was expressed in μmoles/h/g biocatalyst, defined as the amount of the ester (μmoles) synthesized per time unit (1 h) by 1 g of biocatalyst under specific reaction conditions (80 °C or 70 °C, CA:PE at different molar ratio, and 24 h reaction time).

## 3. Results and Discussion

The enzymatic synthesis of CAPE was initiated by performing organic-solvent-based catalytic reactions established from the literature. To optimize the process, key parameters such as reaction temperature, time, and solvent volume were adjusted. The influence of solvents with different polarity on the overall synthesis was also tested. In the second part of our study, we changed traditional solvents with deep eutectic solvent, obtained in a “one-pot” system by using choline chloride (ChCl)-based DES. ChCl is a quaternary ammonium salt, characterized by its low cost, renewability, non-toxicity, and biodegradability, and it is the most commonly used hydrogen bond acceptor in DES systems [36,37]. Several biocatalysts, including native and immobilized enzymes, were thus used to facilitate the esterification process. The evaluated parameters are represented by the ester yield (%) and biocatalyst efficiency (µmol/h/g), essential for assessing the performance of the reaction and the effectiveness of the biocatalyst used.

### 3.1. Enzymatic Synthesis of CAPE in Various Organic Solvents

**Figure 3:**
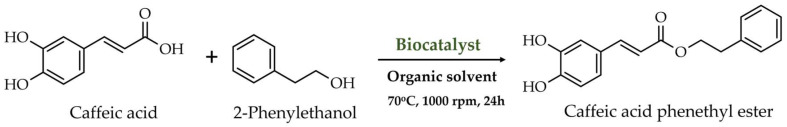
Enzymatic synthesis of CAPE by lipase biocatalysis in different organic solvents.

In the first stage, the synthesis of CAPE was carried out in various organic solvents (acetone, acetonitrile, tetrahydrofuran (THF), Me-THF, n-hexane, and isooctane) through biocatalysis with *A. niger* lipase, at a molar ratio of reactants CA and PE of 1:92 (Figure 3).

**Figure 4:**
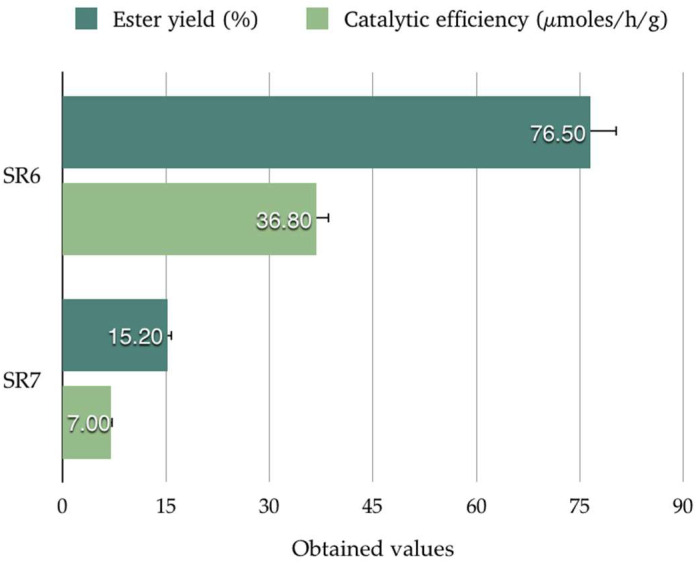
Ester yields (%) and biocatalyst efficiency (µmol/h/g) obtained for SR_6_ and SR7 (in isooctane as solvent).

Chromatographic analysis (HPLC) showed that ester CAPE was obtained exclusively for the solvent that possessed the highest value of log P, only in isooctane, for SR_6_ and SR_7_ (Figure 4). One potential explanation for this phenomenon is the toxicity of polar solvents towards enzymes. It is well established that polar solvents can remove water, which is essential for maintaining the 3-D conformation of the enzyme molecule. This, in turn, can result in a decrease in enzyme activity and, consequently, low reaction yields [38,39,40].

In our study, an ester yield (%) of 76.5 was obtained for reaction SR_6_, with a biocatalyst efficiency of 36.8 µmol/h/g, whereas, for SR_7_, the obtained yield was 15.2%, with a biocatalyst efficiency of 7.0 µmol/h/g. Similar results were obtained by Widjaja et al. [39]. However, for the other reactions, namely, RS_1_, RS_2_, RS_3_, RS_4_, and RS_5_, no ester yield that could be considered significant was obtained. Following these reactions, it was observed that the synthesis of CAPE can be successfully carried out in isooctane, achieving ester yields of over 70%.

### 3.2. Biocatalytic Esterification of CAPE in Deep Eutectic Solvent

**Figure 5:**
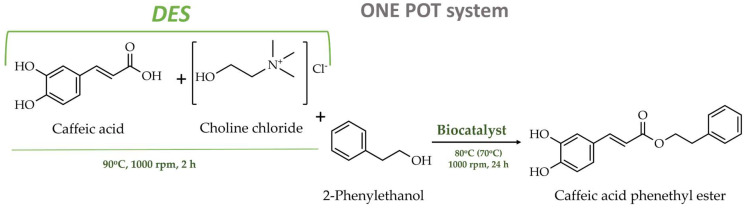
Enzymatic synthesis of CAPE by lipase biocatalysis in deep eutectic solvent.

As mentioned above, the principal aim of this study was to synthesize CAPE in a “green” and non-toxic reaction medium. For this, we synthesized CAPE from caffeic acid (CA) and 2-phenylethanol (PE) in a choline-chloride-based deep eutectic solvent, conducted through a “one-pot” procedure involving biocatalysis with various lipases at a temperature of 80 °C or 70 °C. The schematic representation of this process is depicted in Figure 5.

As caffeic acid (CA) is not soluble in 2-phenylethanol (PE), the deep eutectic solvent was first obtained by mixing CA with choline chloride (ChCl) at a molar ratio of 1:2 for 2 h at 90 °C. This approach enables the use of caffeic acid as both reactant and solvent, eliminating the need for an additional solvent. Subsequent to the formation of DES, the temperature was reduced to 80 °C, or 70 °C when alcohol was incorporated, and a biocatalyst was added. It is acknowledged that the melting point of the produced DES is 67 °C [41]. Consequently, the esterification reaction is unable to occur at temperatures below this point.

**Figure 6:**
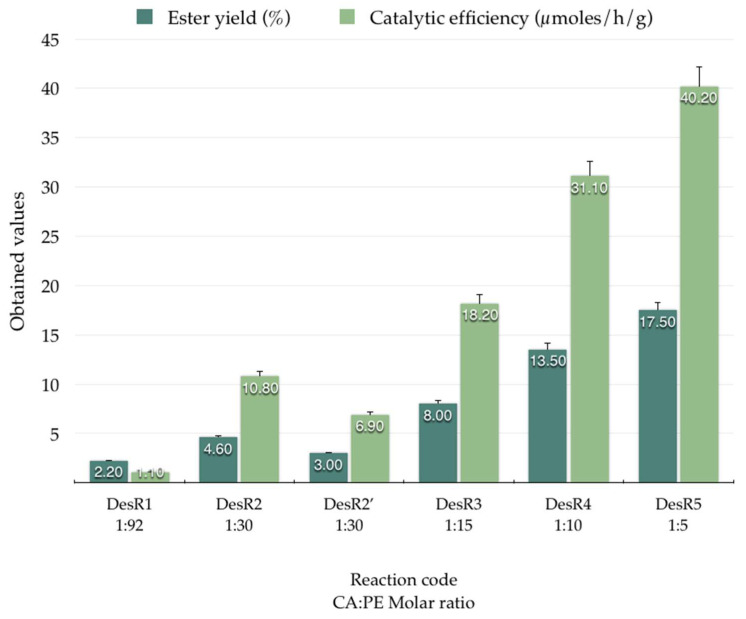
Ester yields (%) and catalytic efficiency (µmoles/h/g) obtained for reactions in DES at 80 °C and different molar ratio of CA:PE, catalyzed by immobilized *A. niger* lipase (AnL-IM).

**Figure 7:**
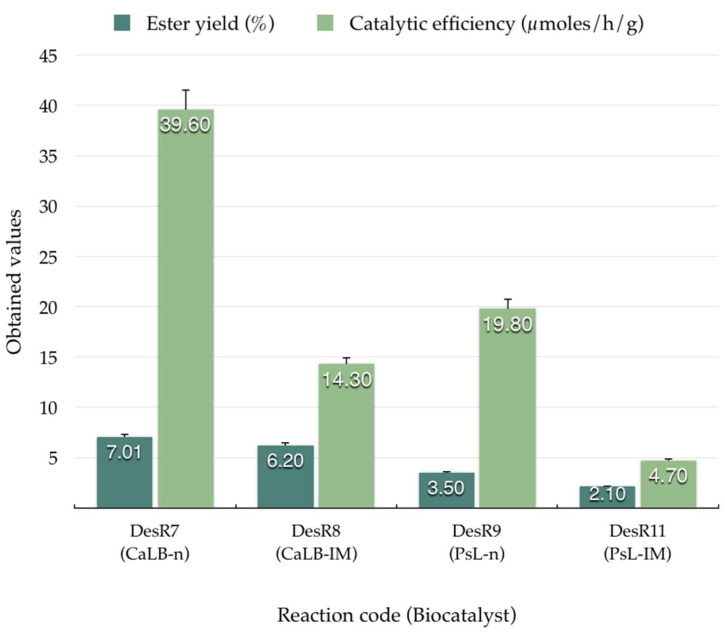
Ester yields (%) and catalytic efficiency (µmoles/h/g) obtained for reactions in DES at 70 °C with the use of different biocatalysts (CaLB-n: *C. antarctica* lipase B native; CaLB-IM: *C. antarctica* lipase B, immobilized; PsL-n: *P. stutzeri* lipase native; PsL-SG2: *P. stutzeri* lipase sol–gel, immobilized).

The molar ratio of caffeic acid (CA) and 2-phenylethanol (PE) is a critical parameter influencing the enzymatic synthesis of caffeic acid phenethyl ester (CAPE). Studies have demonstrated that optimizing this ratio can significantly enhance reaction yields. For instance, Widjaja et al. reported that, in isooctane, the optimal molar ratio of CA:PE was 92:1 [39], whereas, in ionic liquids (ILs), when the molar ratio was 30:1, the conversion rates were lower [42]. Further optimization using a response surface methodology identified a molar ratio of 27.1:1 in ILs as optimal, achieving a conversion yield of 96.6% [43]. These findings underscore the importance of carefully adjusting the substrate molar ratio to enhance the efficiency of CAPE synthesis, as excessive amounts of phenethyl alcohol can lead to enzyme inactivation, while insufficient amounts may limit ester formation. In this scope, various molar ratios of caffeic acid (CA) to phenethyl alcohol (PE) were further tested. The obtained results (reaction ester yields and catalytic efficiency) are presented in Figure 6 and Figure 7, respectively.

As seen in Figure 6, for the DesR_1_ reaction, using AnL-IM as a biocatalyst with an enzyme mass of 50 mg (CA:PE molar ratio of 1:92), the reaction yield was 2.2% and the biocatalyst efficiency was 1.1 µmol/h/g. The DesR_2_ reaction, performed with 50 mg enzyme and a molar ratio of 1:30, indicated a yield of 4.6%, with a biocatalyst efficiency of 10.8 µmol/h/g. In the case of DesR_2‘_, with an enzyme mass of 100 mg and a molar ratio of 1:30, the yield was 3.0% and the biocatalyst efficiency was 6.9 µmol/h/g. For the DesR_3_ reaction with an enzyme mass of 50 mg (CA:PE molar ratio of 1:15), a reaction yield of 8.0% was obtained, and the efficiency of the biocatalyst was 18.2 µmol/h/g. In the case of the DesR_4_ reaction, with 50 mg of enzyme and a molar ratio of 1:10, a yield of 13.5% was obtained, and the efficiency of the biocatalyst increased to 31.1 µmol/h/g. For the DesR_5_ reaction, the highest values were obtained, with the yield being increased to 17,5% and the efficiency of the biocatalyst being 40.2 µmol/h/g. In the case of the DesR_3′_ and DesR_6_ reactions, no homogeneous complex was formed; thus, the biocatalyst efficiency was not calculated.

The 1:5 molar ratio of CA:PE both demonstrated satisfactory homogeneity for the synthesis process and gave the highest ester yield of 17.5%. These results underscore the significance of identifying an optimal molar ratio, a critical factor in optimizing reaction conditions and maximizing product yield.

Following the determination of the optimal CA:PE molar ratio, a reduction in the temperature of the reaction system to 70 °C was implemented. However, in the synthesis with AnL-IM, no homogeneous mixture was obtained, thereby impeding the progression of the reaction. Consequently, the decision was made to assess the efficacy of other native and immobilized lipases at this temperature. Results are shown in Figure 7.

The DesR_7_ reaction, utilizing CaLB-n as the biocatalyst and an enzyme mass of 20 mg, exhibited a modest yield of 7.01%, though it demonstrated a notable efficiency of 39.6 μmol/h/g. Results were consistent with those reported by Fischer et al. [18] and Wang et al. [44]. In the case of the DesR_8_ reaction, using CaLB-IM with 50 mg of enzyme, the yield decreased slightly to 6.2% and the biocatalyst efficiency was lower at 14.3 μmol/h/g. The DesR_9_ reaction, with PsL-n (20 mg), had an even lower yield of 3.5%, and instead gave a moderate catalytic efficiency of 19.8 μmol/h/g. For the DesR_10_ reaction, using our sol–gel enzymatic preparate PsL-SG1 (50 mg) resulted in the absence of an ester yield because no complex was formed in the reaction process. This consequently led to its non-appearance in Figure 7. This could be due to the fact that the polymeric sol–gel matrix created around the enzyme was too tight and the substrate could not access the enzyme. In contrast to DesR_10_, the reaction of DesR_11_ with the sol–gel enzymatic preparate PsL-SG_2_ (50 mg) gave an ester yield, but it was quite low at only 2.1%, and a catalytic efficiency value of only 4.7 μmol/h/g. This could be due to the fact that the PsLSG_2_ biocatalyst is obtained with a 3-glycidyloxypropyl group silane, which is much bulkier than the octyl group from the other sol–gel matrix, and probably created a much more accessible polymer matrix for the caffeic acid.

The findings of this study demonstrate a considerable impact of the biocatalyst nature, mass, and the molar ratio between CA and PE on the ester yield and catalytic efficiency.

### 3.3. Biocatalytic Esterification of CAPE in Deep Eutectic Medium with Water Addition

**Figure 8:**
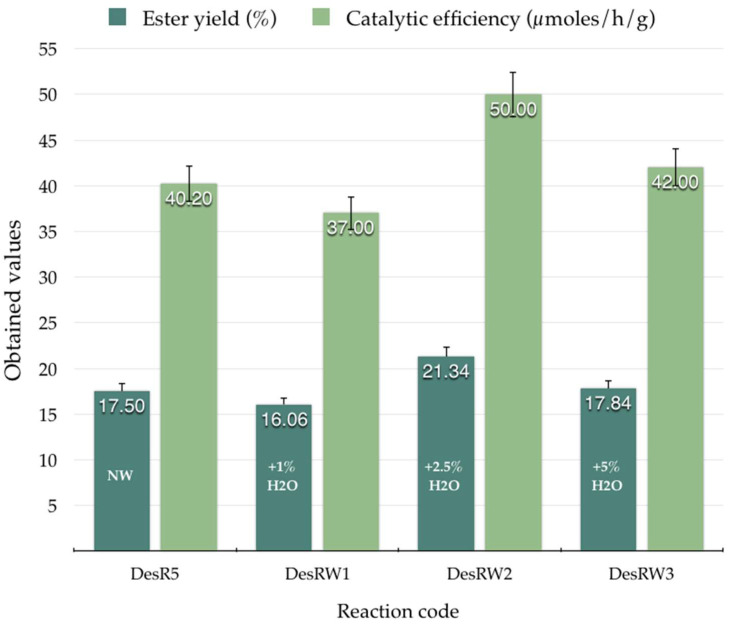
Ester yields (%) and catalytic efficiency (µmoles/h/g) obtained for reactions in DES at 80 °C with water addition (1%, 2.5%, and 5%), no water added (NW), and *A. niger* lipase, immobilized (AnL-IM), as biocatalyst.

In the initial phase of the study, a series of molar ratios and different biocatalysts were investigated. The highest ester yield was achieved with a CA:PE molar ratio of 1:5, using AnL-IM as biocatalyst. In order to enhance the homogeneity of the reaction medium and potentially improve the reaction efficiency, esterification was further investigated by introducing water into the system (1%, 2.5%, and 5% water loading) while maintaining the same biocatalyst (AnL-IM) and the same molar ratio of CA:PE at 1:5, as in the case of DesR_5_. The obtained results are presented in Figure 8.

The results for the synthesis of CAPE in a eutectic solvent using 50 mg immobilized *A. niger* lipase (AnL-IM) and different degrees of water loading after 24 h of reaction at 80 °C were as follows: For DesRW₁, with a water loading of 1%, the ester yield was 16.06%, and the efficiency of the biocatalyst was 37.0 μmol/h/g. Increasing the water loading to 2.5%, in the case of DesRW_2_, resulted in a significant increase in both ester yield (21.34%) and the biocatalyst efficiency (50.0 μmoles/h/g). This suggests that higher water content improves the catalytic environment, possibly by increasing enzyme flexibility or reactant solubility. For DesRW_3_, at the highest water loading (5%), both ester yield and catalytic efficiency were reduced, to 17.84% and 42.0 μmoles/h/mg, respectively. This decline may signify that excess water disrupts the eutectic solvent system or adversely impacts enzyme activity. The results highlight that a moderate degree of water loading (2.5%) optimizes both the ester yield and efficiency of the biocatalyst, while lower or higher water contents compromise performance.

Table 5 shows examples of biocatalytic CAPE synthesis reactions, carried out in organic solvents, with different catalysts, reported by other groups in comparison with our results.

From this table it is evident that the synthesis of CAPE demonstrates yield variations across different methods, with enzymatic esterification in isooctane and a biphasic system involving ionic liquids achieving a 90% reaction yield, and esterification using SOCl_2_ reaching an 86% yield. In contrast, the DCC-condensed reaction yielded only 38%, highlighting inefficiencies under milder conditions. Enzymatic synthesis in the ionic liquid [Emim][Tf2N] resulted in a yield of 63.75%, reflecting moderate efficiency. Fischer et al. [18] reported a yield of 72% in deep eutectic solvent (DES) synthesis with Amberlyst 15, offering a balance between efficiency and environmental sustainability under reduced reaction temperatures and mild conditions, emphasizing its industrial relevance.

In line with this approach, our study also achieved the “one-pot” synthesis of CAPE in DES via enzymatic catalysis, employing a molar ratio of CA:PE of 1:5 and AnL-IM as the biocatalyst, with only water addition. The reaction was conducted at temperature of 80 °C for 24 h, yielding over 21%. Additionally, various enzymes, both native and immobilized, were tested, demonstrating biocatalyst efficiencies exceeding 49 µmole/h/g. It is evident that the yields obtained in our study are modest; however, it should be noted that the ester was synthesized in a green solvent prepared in a “one-pot” process with choline-chloride-based DES, caffeic acid (CA), and phenethyl alcohol (PE), with water being the only additive introduced during the second phase of the study. Furthermore, the enzymatic synthesis was successfully carried out at a relatively high temperature, which, while challenging for optimal lipase functionality, was necessary to ensure the homogeneity of the reaction mixture.

### 3.4. Future Prospects

Future perspectives include further optimization of the enzymatic synthesis process in eutectic solvent to improve the yield and efficiency of CAPE production. The application of this one-pot reaction system in eutectic solvents to other bioactive molecules of pharmaceutical or food interest could open new opportunities for biotechnology, reducing the dependence on conventional organic solvents. It is also relevant to explore the stability and enzymatic activity in different types of eutectic solvents, which could contribute to the creation of new green synthetic synthesis methods. From an economic point of view, optimizing the process not only validates the feasibility of producing the ester in sustainable systems, but also, integrated at an industrial scale, it could lead to the development of more sustainable processes with applications in the production of nutraceuticals, cosmetics, or therapeutic agents [48,49]. Furthermore, the investigation of the antioxidant and antimicrobial potential of the product obtained in different formulations could support the validation of its commercial applicability in several industries [50].

## 4. Conclusions

The enzymatic synthesis of caffeic acid phenethyl ester (CAPE) was successfully achieved using both conventional solvents and eutectic solvents, demonstrating the versatility of this approach. The obtained results showed a significant influence of the enzyme and the molar ratio between CA and PE on the ester yield and catalytic efficiency. The “one-pot” synthesis of CAPE in a choline-chloride-based deep eutectic solvent system assured a significant ester yield of 17.5%, and the efficiency of the biocatalyst was 40.2 µmoles/h/g (DesR_5_). Notably, the DES system with a water addition of 2.5% (DesRW_2_) indicated the highest ester yield and biocatalyst efficiency, 21.34% and 50.0 μmoles/h/g, respectively. The reaction conditions, optimized at 80 °C and a reaction time of 24 h, underlined the efficiency of the enzymatic process, with only 50 mg of biocatalyst per synthesis ensuring consistent and reproducible results.

## Figures and Tables

**Table 1:** Significant characteristics of deep eutectic solvents.

DES Property	Detailed Characteristics	Reference
Biodegradability	biodegradable, with minimal environmental impact compared to traditional solvents.	[20,21]
Non-Toxicity	less toxic to humans and the environment, making them safer for various applications.	[21,22]
Renewable Components	many DES are prepared from renewable raw materials, reducing reliance on petrochemical resources.	[23]
Cost Effectiveness	can be synthesized easily and inexpensively, often using readily available components.	[24]
Energy Efficiency	the synthesis of DES does not require high-energy inputs, leading to reduced operational costs.	[25]
Reduced Volatility	exhibit low vapor pressures, minimizing emissions and the risk of air pollution.	[26]
“2-in-1” Concept	simultaneously act as both solvents and substrates, eliminating the need for additional solvents and enhancing atom efficiency.	[20]
Chemical Property Control	allow for tuning of viscosity, pH, and enzyme activity by varying their components, making them highly customizable for specific reactions.	[20]

**Table 2:** Biocatalytic esterification of CAPE in different solvents using as biocatalyst *A. niger* lipase (AnL-IM).

	Reaction Name	Solvent Name	Solvent log P	Solvent Volume (mL)	Enzyme (mg)	Temperature (°C)
1.	SR_1_	acetone	−0.24	10	50	70
2.	SR_2_	acetonitrile	−0.33			
3.	SR_3_	THF	0.49			
4.	SR_4_	Me-THF	1.26			
5.	SR_5_	n-hexane	3.5			
6.	SR_6_	isooctane	4.5			
7.	SR_7_	isooctane	4.5	5		

**Table 3:** Biocatalytic esterification of CAPE in deep eutectic solvent.

	Reaction Name	^1^ CA:^2^ ChCl Molar Ratio	CA:^3^ PE Molar Ratio	Biocatalyst Name	Enzyme (mg)	Temperature (°C)
1.	DesR_1_	1:2	1:92	^4^ AnL-IM	50	80
2.	DesR_2_		1:30	AnL-IM	50	
3.	DesR_2′_		1:30	AnL-IM	100	
4.	DesR_3_		1:15	AnL-IM	50	
5.	DesR_3′_		1:15	AnL-IM	30	
6.	DesR_4_		1:10	AnL-IM	50	
7.	DesR_5_		1:5	AnL-IM	50	
8.	DesR_6_		1:5	AnL-IM	50	70
9.	DesR_7_		1:5	^5^ CaLB-n	20	
10.	DesR_8_		1:5	^6^ CaLB-IM	50	
11.	DesR_9_		1:5	^7^ PsL-n	20	
12.	DesR_10_		1:5	^8^ PsL-SG1	50	
13.	DesR_11_		1:5	^9^ PsL-SG2	50	

^1^ caffeic acid; ^2^ choline chloride; ^3^ 2-Phenylethanol; ^4^ *A. niger* lipase (immobilized); ^5^ *C. antarctica* lipase B (native); ^6^ *C. antarctica* lipase B (immobilized); ^7^ *P. stutzeri* lipase (native); ^8^ *P. stutzeri* lipase (immobilized with OcTMOS:TMOS in 1:1 molar ratio); ^9^ *P. stutzeri* lipase (immobilized with 3-GOPrTMOS:TMOS in 1:1 molar ratio).

**Table 4:** Biocatalytic esterification of CAPE in DES with water addition.

	Reaction Name	^1^ CA:^2^ ChCl Molar Ratio	^1^ CA:^3^ PE Molar Ratio	Biocatalyst Name	Enzyme (mg)	Water Addition (%)	Temperature (°C)
1.	DesRW_1_	1:2	1:5	^4^ AnL-IM	50	1.0	80
2.	DesRW_2_		1:5	AnL-IM	50	2.5	
3.	DesRW_3_		1:5	AnL-IM	50	5.0	

^1^ caffeic acid; ^2^ choline chloride; ^3^ 2-Phenylethanol; ^4^ *A. niger* lipase (immobilized).

**Table 5:** Examples of CAPE synthesis in different reaction media.

Synthesis Method	Catalyst	Reaction Yield (%)	Temperature (°C)	Reaction (h)	Reference
Synthesis in DES	Amberlyst 15	72	80	14	[18]
Esterification in [Emim][Tf2N]	Novozym 435	63.7	84	120	[45]
Esterification using DCC as condensing reagent	Dicyclohexyl carbodiimide (DCC)	38	25	8	[46]
Esterification using SOCl_2_ and phenethyl alcohol	Thionyl chloride (SOCl_2_)	86	100 (reflux)	2	[47]
Esterification in isooctane	Novozym 435	≥90	70	48	[39]
Transesterification in a biphasic system ([Bmim][Tf2N] and cyclohexane with TOPO)	Novozym 435 + TOPO (30 g/L)	≥90	76	59	[17]

## Data Availability

Data are contained within the article.
